# MALAT1: a druggable long non-coding RNA for targeted anti-cancer approaches

**DOI:** 10.1186/s13045-018-0606-4

**Published:** 2018-05-08

**Authors:** Nicola Amodio, Lavinia Raimondi, Giada Juli, Maria Angelica Stamato, Daniele Caracciolo, Pierosandro Tagliaferri, Pierfrancesco Tassone

**Affiliations:** 1 Department of Experimental and Clinical Medicine, Magna Graecia University, Viale Europa, 88100 Catanzaro, Italy; 2 IRCSS Rizzoli Orthopedic Institute, Bologna, Italy; 3 Innovative Technology Platforms for Tissue Engineering, Theranostic and Oncology, Rizzoli Orthopedic Institute, Palermo, Italy

**Keywords:** MALAT1, Long non-coding RNA, lncRNA, Non-coding RNA, Epigenetics, Experimental therapeutics

## Abstract

The deeper understanding of non-coding RNAs has recently changed the dogma of molecular biology assuming protein-coding genes as unique functional biological effectors, while non-coding genes as junk material of doubtful significance. In the last decade, an exciting boom of experimental research has brought to light the pivotal biological functions of long non-coding RNAs (lncRNAs), representing more than the half of the whole non-coding transcriptome, along with their dysregulation in many diseases, including cancer.

In this review, we summarize the emerging insights on lncRNA expression and functional role in cancer, focusing on the evolutionary conserved and abundantly expressed metastasis-associated lung adenocarcinoma transcript 1 (MALAT1) that currently represents the best characterized lncRNA. Altogether, literature data indicate aberrant expression and dysregulated activity of MALAT1 in human malignancies and envision MALAT1 targeting as a novel treatment strategy against cancer.

## Background

### Long non-coding RNAs (lncRNAs): classification and mechanisms of action

Deep analysis of the human transcriptome has estimated that while three quarters of the human genome is actively transcribed, only the 3% is represented by protein-coding genes. While such a huge non-coding genome was considered transcriptional noise and neglected until the last decade, its biological relevance is nowadays well-acknowledged and represents a matter of intense investigation.

Different types of classification for non-coding RNAs (ncRNAs) have been so far adopted. Based on their length, ncRNAs can be classified into short (< 200 nucleotides) or lncRNAs (> 200 nucleotides) [1]. LncRNAs are the most abundant class of ncRNAs involved in important biological processes, such as epigenetic control of gene expression, promoter-specific transcriptional regulation [2], X chromosome inactivation [3, 4], genomic imprinting [5, 6], and cell differentiation and development [7]; consistent with a role in these processes, it is not surprising that lncRNAs are found dysregulated in different human diseases, including cancer [8]. Overall, these RNA molecules do not show protein-coding potential, localize predominantly to the nucleus, and are generally less expressed than protein-coding genes, while displaying more tissue-specific expression patterns; moreover, lncRNAs generally undergo splicing and can be polyadenylated [1, 9].

Based on molecular function, lncRNAs can be distinguished in the following:

- lncRNAs signal that regulate the transcription by interacting with transcription factors (TFs), and activate or repress their activity;
- lncRNAs decoy that displace TFs and other proteins from chromatin of which they are negative regulators;
- lncRNAs scaffold that bind multiple proteins and facilitate the formation of ribonucleoprotein complexes;
- lncRNAs guide, acting as guidance molecules for chromatin-modifying protein complexes to target genes [10].

While cis-acting RNAs are limited to the site of synthesis and operate on one or more genes on the same chromosome, trans-acting RNAs move from the site of synthesis and exert their function at larger distances or even on other chromosomes [11]. Moreover, lncRNAs can modulate the mRNA life cycle in the cytoplasm through different mechanisms, including the following:

- mRNA stability regulation by Staufen 1 (STAU1)-mediated decay, in which base-pairing of ALU elements located in the lncRNA and the target mRNA creates a double-stranded STAU1-binding site that can direct exosome mediated RNA degradation [12];
- RNA splicing regulation by direct association between lncRNAs and splicing factors;
- RNA editing regulation with activation of ADAR enzymes induced by the interaction of antisense lncRNA and sense mRNA [11];
- miRNA sponges that contain multiple binding sites for one or more miRNAs and regulate target mRNA expression by sequestering miRNAs away from their mRNA targets [13, 14].

LncRNAs can be also classified according to their genomic location with respect to the nearest protein-coding genes. In this regard:

- Long intergenic non-coding RNAs (lincRNAs) are ncRNAs which do not lie close to protein-coding genes;
- Sense lncRNAs are on the same strand of protein-coding genes and are transcribed in the same direction;
- Antisense lncRNAs lie on the opposite strand of protein-coding genes with which they overlap (if the overlap is partial, lncRNAs are defined natural antisense transcripts);
- Intronic antisense lncRNAs and bidirectional lncRNAs that locate on the other strand with respect to protein-coding genes and are transcribed in the opposite direction [15, 16].

Several lncRNA databases providing relevant information on lncRNA structure and functional role are currently available. Noteworthy, LNCipedia annotates human lncRNA sequences and structures [17], while lncRNAdb contains information about lncRNA biological functions and expression in different biological systems [18]. The GENCODE consortium includes human genome annotation for the ENCODE project [19], while the NONCODE database (v3.0 and 4.0) has extended available information on lncRNA cellular localization, function and expression to the cancer setting [20, 21]. For in-depth information on lncRNA databases, we recommend to the readers more specialized reviews [22].

### Cancer-related mechanisms of lncRNA dysregulation

LncRNAs have emerged as novel master regulators of initiation, progression, and response to therapy in a wide variety of solid tumors and hematological malignancies. Compelling evidence supports aberrant patterns of expression of lncRNAs in cancer [16], which may occur through various mechanisms, summarized in Table 1.

**Table 1:** Mechanisms involved in dysregulated expression of the most relevant cancer-associated lncRNAs

Mechanism	lncRNA	Effect on lncRNA expression	Reference
Chromosomal deletion	DLEU1/2	Downregulation	[23]
Chromosomal amplification	PVT1	Upregulation	[28]
Polymorphisms in enhancer regions	HOTAIR	Upregulation	[29]
CpG methylation	KIAA0495	Downregulation	[32]
CpG methylation	MEG3	Downregulation	[34]
p53	lincRNA-p21	Upregulation	[40]
p53	PINT	Upregulation	[41]
p53	H19	Downregulation	[42]
MYC	MYCLo1/2	Upregulation	[46]
MYC	PCAT1	Upregulation	[50]
MYC	H19	Upregulation	[55]
MYC	HOTAIR	Upregulation	[29]
Notch1	LUNAR	Upregulation	[58]
Notch1	NALT	Upregulation	[59]
ER	DSCAM-AS1	Upregulation	[63]
ER	NEAT1	Upregulation	[64]

#### Genomic alterations

Deletions or amplification of gene loci may be responsible of abnormal expression of lncRNAs in cancer. Deleted in leukemia 1 (DLEU1) and 2 (DLEU2) are two lncRNAs whose genes map in a critical region at chromosome 13q14.3 deleted in more than 50% of chronic lymphocytic leukemia (CLL) patients [23], and hosting miR-15a and 16–1, a miRNA tumor suppressive cluster [24]. Mice deleted for the entire minimal deleted region within 13q14, comprising DLEU2 gene, developed clonal B cell proliferations resembling human CLL [25, 26]. LncRNA expression profiling of the major forms of plasma cell dyscrasias highlighted significant downregulation of DLEU2 in patients carrying del13 [27].

Amplification at 8q24 locus, which correlated with reduced overall survival of breast and ovarian cancer patients, was responsible of increased expression of the lncRNA PVT1, whose in vitro inhibition triggered apoptosis and reduced survival of cancer cell lines [28]. The presence of tumor-associated single nucleotides polymorphisms (SNPs) within enhancer regions of certain lncRNAs, including HOTAIR, may also drive their dysregulated expression and was found to correlate with high risk of tumor development [29].

#### Epigenetic alterations

Epigenetic modifications account for aberrant expression of short ncRNAs [30], but are also implicated in lncRNA dysregulation. Zhang and colleagues integrated multi-omics data to assess alterations of lncRNA methylation in breast invasive carcinoma, highlighting abnormally methylated lncRNAs involved in several hallmarks of cancers [31]. In multiple myeloma (MM) cells, the methylation of promoter-associated CpG islands triggered downregulation of KIAA0495, a lncRNA transcribed from chromosome 1p36 [32]; CpG island hypermethylation also downregulated the KIAA0495/p53-dependent apoptotic modulator PDAM, which in turn established a drug resistant phenotype in glioma cells [33].

Aberrant promoter methylation of the imprinted lncRNA gene MEG3 was described in acute myeloid leukemia and myelodysplastic syndromes patients, where MEG3 methylation status negatively correlated with overall survival [34]; MEG3 promoter hyper-methylation has been also reported in MM [35] and in esophageal squamous cell carcinoma (ESCC) [36].

#### P53-dependent regulation

Several factors involved in cellular homeostasis may regulate lncRNA expression. For instance, similarly to miRNAs [37], some lncRNAs have been found associated with the p53 tumor suppressor pathway [38, 39]. P53 bound to and transcriptionally activated the promoter of lincRNA-p21 that resides 15Kb upstream of the gene encoding the cell cycle regulator CDKN1A; in turn, lincRNA-p21 mediated p53-dependent induction of cell death by repressing gene expression partly through interaction with the heterogeneous nuclear ribonucleoprotein k (hnRNP-k) [40]. Marìn-Bejar et al. showed that p53 activates PINT, a lincRNA downregulated in colon tumors, whose enforced expression decreased in vitro proliferation of cancer cell lines [41]; moreover, p53 may also repress the promoter of the H19 lncRNA [42], a maternally imprinted gene overexpressed in fetal tissues and silenced after birth, initially described as tumor suppressor [43], but also endowed with oncogenic properties in certain tissues. Accordingly, high levels of H19 prompted tumorigenesis by negatively regulating p53 activity in gastric cancer [44], since miR-675, the mature product of H19, can directly target and down-regulate p53 [45].

#### MYC-dependent regulation

c-MYC oncogene can also account for lncRNA regulation in tumor cells. MYCLos are MYC-regulated lncRNAs positively mediating MYC-mediated tumorigenesis, whose promoters are highly enriched for Myc-binding sites. In the nucleus, MYCLo-1 and MYCLo-2 physically interact with HuR and hnRNPK RNA-binding proteins respectively, finally repressing CDKN1A and CDKN2B transcription [46–48]. RNA-Seq analysis of the prostate cancer transcriptome identified 121 unannotated ncRNA transcripts, of which PCAT-1 (prostate cancer-associated transcript 1) is a sense lncRNA localized upstream of the MYC gene at the 8q24 genomic region [49]. Prensner et al. showed that prostate cell proliferation promoted by PCAT-1 was c-MYC dependent; in turn, PCAT-1 increased MYC 3′UTR activity and exerted a protective effect on tumor cells by abolishing miR-34a-dependent targeting of c-MYC [50–52]. c-MYC also binds to the E-boxes located in the H19 gene promoter, thus facilitating histones acetylation and transcription of the H19 gene [53–56].

The human PVT1 lncRNA is located on chromosome 8 telomeric to the c-Myc gene and is frequently involved in the translocations occurring in variant Burkitt’s lymphomas and murine plasmacytoma; PVT1 presents two MYC-binding sites in its promoter, which are bound and transactived by c-MYC [57].

c-MYC-dependent transcriptional activation through E-box located upstream of the transcription start site also accounted for elevated expression of the oncogenic lncRNA HOTAIR in human cancer [29].

#### Notch-1-dependent regulation

Notch1/RBPJk-binding sites have been detected in several lncRNA promoters. Trimarchi et al. showed that Notch mediates the transcriptional activation of several lncRNAs in T-acute lymphoblastic leukemias (T-ALL). The leukemia-induced non-coding activator RNA LUNAR1 was discovered as Notch-regulated oncogenic lncRNA in T-ALL cells, which promoted cancer cell proliferation by activation of IGF-1 signaling [58]. The lncRNA RP11-611D20.2, also named NALT (Notch1 associated lncRNA in T-ALL), was found located near Notch1 gene, and its overexpression sustained in vitro and in vivo T-ALL growth [59].

#### ER-mediated regulation

Estrogen receptor (ER) signaling was shown to be involved in tumorigenesis through the regulation of a subset of lncRNAs. Deep sequencing analysis following estrogen stimulation [60–62] highlighted 133 lncRNAs associated with luminal histotype of breast cancer, of which DSCAM-AS1 was found predominantly expressed in ERα^+^ cases, but not in ERα^−^ and in pre-neoplastic lesions. DSCAM-AS1 knockdown recapitulated the effects caused by ERα silencing, as the arrest of cancer cell growth and induction of EMT [63]. In the study by Chakravarty et al., the nuclear-enriched abundant transcript 1 (NEAT1) was among the top ERα-regulated lncRNAs in prostate cancer as compared with benign prostate tissues; in prostate cancer cell lines, ERα overexpression upregulated NEAT1 [64], which in turn activated the transcription of genes involved in prostate cancer progression [64].

MALAT1 is the first discovered and most widely investigated lncRNA, in terms of both functional activity and therapeutic potential for cancer treatment [65].

Different molecular mechanisms have been implicated in MALAT1 dysregulation in human cancer, and will be in-depth analyzed in the next paragraphs.

### MALAT1 expression and regulation

The transcript of MALAT1 gene, also known as NEAT2 for nuclear-enriched abundant transcript 2, consists of > 8000 nucleotides as described by Ji et al. [65]. Although early reports provided evidence of its association with metastasis in early-stage non-small cell lung cancer (NSCLC) patients, subsequent studies reported that MALAT1 is extremely abundant and widely conserved among 33 mammalian species [65, 66]. Later, MALAT1 was reported to be a highly abundant nuclear transcript localized to the nuclear speckles, a nuclear domain for storage and/or the sites of pre-mRNA splicing; computational and biochemical studies then confirmed interaction between MALAT1 and serine- and arginine-rich (SR) proteins involved in splicing regulation, or with spliceosomal proteins [67].

MALAT1 is transcribed by RNA polymerase II from the human chromosome 11q13. Its biogenesis relies on the tRNA processing machinery, by a regulatory mechanism that allows a single locus to produce two non-coding RNAs that localize to different subcellular compartments and have distinct functions. In detail, the tRNA-like structure at 3′ end of the primary transcript is cleaved by tRNA endonucleases RNase P and RNase Z, generating both a long transcript localizing to nuclear bodies with a short poly(A) tail-like moiety, and a smaller 61-nucleotides MALAT1-associated small cytoplasmic RNA (mascRNA) that is exported to the cytoplasm. The mascRNA folds similar to a tRNA cloverleaf secondary structure, allowing it to be recognized by several members of the canonical tRNA processing machinery [68, 69].

MALAT1 half-life (9–12 h) is longer than other lncRNAs, probably for the presence of a triple helix structure at its 3′ end that confers stability by engaging a downstream A-rich tract [70].

Noteworthy, the nuclear methyltransferase-like protein 16 (METTL16) was shown to interact with the MALAT1 triple-helix in vitro and in vivo [71] and to catalyze the N(6)-methyladenosine (m6A) modification, a reversible post-transcriptional modification of MALAT1 hairpin that facilitates the accessibility of a U5-tract for the binding to hnRNP C, a nuclear protein necessary for pre-mRNA processing [72].

A relevant interactor of MALAT1 is TAR DNA-binding protein TDP43 located in the mouse brain. TDP43 is a predominantly nuclear protein that regulates transcription, alternative splicing and RNA stability [73]. In human brains from subjects with fronto-temporal lobar degeneration having TDP-43 inclusions (FTLD-TDP), binding of MALAT1 to TDP43 increased, thus suggesting a link between MALAT1 and neurodegenerative diseases [74].

MALAT1 undergoes a tight transcriptional control in tumor cells. Several transcription factors can regulate MALAT1 transcription, either in a positive manner, as SP1, SP3 [75], β-catenin [76], HIF1α [77] and HIF2α [78], c-MYC [79], Yes-associated protein 1 (YAP1) [80], NRF1 [81], or in a negative fashion, like p53 [82] and SOX17 [83]. Moreover, hormones, such as oxytocin [84], or growth factors, such as TGF-β [85], can transcriptionally induce MALAT1 leading to tumor growth.

The Drosha-DGCR8 complex, a component of miRNA biogenesis machinery, was found to interact with the 5′ end of MALAT1 and to regulate its stability [86]. The involvement of miRNAs in MALAT1 expression regulation was further corroborated by the study of Leucci et al., who showed that depletion of Ago2 stabilized MALAT1, thus suggesting that miRNAs play a role in MALAT1 modulation; accordingly, miR-9 was shown to target MALAT1 for degradation in the nucleus by directly binding to two miRNA-binding sites within MALAT1 sequence [87]. Furthermore, Ago2-dependent post-transcriptional regulation of MALAT1 by miR-101 and miR-217 significantly impaired proliferation, migration, and invasion of Esophageal Squamous Cell Carcinoma Cells (ESCC) cells, highlighting the MALAT1-dependent tumor suppressor role of these miRNAs [88].

A massive parallel sequence analysis of small RNAs intriguingly identified a number of MALAT1-derived miRNA-like molecules, whose functions is yet to be determined [86].

Epigenetic histone modifications may also regulate MALAT1. For instance, Jumonji C-domain-containing protein JMJD1A, a H3K9 histone demethylase, was found to bind to MALAT1 promoter, and to demethylate histone H3 at lysine 9 (H3K9), thus leading to MALAT1 upregulation [89, 90]. KMD3A, another H3K9 demethylating enzyme, was found to induce MALAT1 expression in MM cells that contributed to the establishment of hypoxic niches supporting tumor growth [91]. Conversely, lysine-specific demethylase 5B KDM5B suppressed MALAT1 expression via miR-448 upregulation that directly targeted MALAT1 [92].

**Fig. 1:**
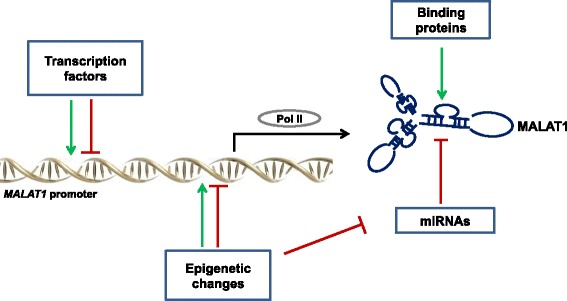
Mechanisms of MALAT1 regulation. MALAT1 expression can be positively or negatively affected by transcription factors, epigenetic changes (histone or CpG methylation/demethylation), miRNAs and binding proteins stabilizing the triple helix, such as METTL16

A graphic overview of the molecular mechanisms underlying MALAT1 expression is provided in Fig. 1.

### MALAT1 molecular functions

**Fig. 2:**
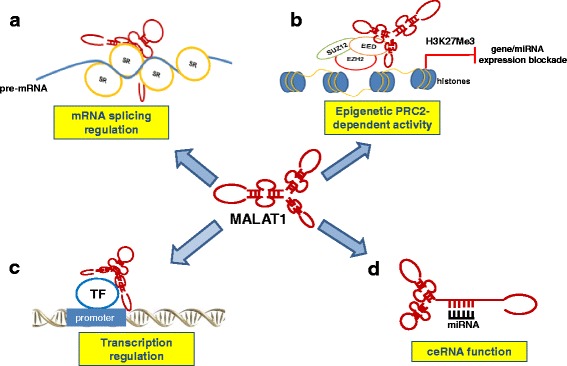
MALAT1 functions in human cancer. **a**: MALAT1 can affect mRNA transcription by regulating splicing of pre-mRNAs through interacting with and regulating phosphorylation of serine- and arginine-rich (SR) proteins into nuclear speckles. **b**: MALAT1 interacts with PRC2 components EZH2, SUZ12, and EED and reduces target gene or miRNA expression by promoting trimethylation of histone H3 at lysine 27 (H3K27me3). **c**: MALAT1 can affect mRNA transcription by facilitating transcription factor (TF) binding to promoter of target genes. **d**: MALAT1 can sequestrate miRNAs acting as a sponge, thus activating the expression of miRNA targets

To date, three major functions have been attributed to MALAT1 (see Fig. 2 for a graphic overview). The contribution of each function to the tumorigenic activity of MALAT1 within specific tumor types will be also discussed in the paragraph “MALAT1 role in cancer”.

#### Alternative splicing

Regulation of alternative splicing is the first identified function for MALAT1. As mentioned before, MALAT1 was found localized in the nucleus, and specifically enriched at nuclear speckle periphery [93]; here, MALAT1 interacted with SR proteins, such as SRSF1, SRSF2, and SRSF3, and its depletion impaired the phosphorylation and the expression pattern of SR proteins, ultimately affecting pre-mRNAs alternative splicing [94] (Fig. 2a); interestingly, silencing of splicing factors induced MALAT1 relocalization from speckles to nucleoplasm [94, 95]. The pro-tumorigenic activity of SRSF1 has been recently established [96], and a whole genome analysis has identified multiple mutations in SRSF1-binding sites of MALAT1, pointing to a role for SRSF1/MALAT1 interaction in cancer pathogenesis [97].

Regulation of alternative splicing by MALAT1 is however under debate, since nuclear speckle components and alternative splicing are not impaired in mouse embryonic fibroblasts generated from MALAT1 knockout mice, thus indicating that MALAT1 is not essential for splicing regulation under physiological conditions [98].

#### Transcriptional regulation

Preliminary evidence of MALAT1 involvement in transcriptional regulation was supported by colocalization of serine-2 phosphorylated RNA polymerase II in nuclear speckle compartment. Later, MALAT1 was reported to interact with unmethylated Pc2, a component of polycomb-repressive complex 1, together with the lncRNA TUG1.

The physical relocation of growth control genes from polycomb bodies is modulated by the methylation/demethylation of Polycomb 2 (Pc2), a component of the polycomb repressive complex 1. MALAT1 functions as a molecular scaffold that allows gene expression during serum stimulation by promoting the interaction among non-methylated Pc2, E2F TF, activation-associated histone markers, and the transcriptional coactivator complex [99]. Consistently, MALAT1 actively interacts with transcribed gene loci and controls their expression, as demonstrated by MALAT1 interaction with the 3′ end of the gene body and overlapping with H3K36me2 peaks, a marker of active transcriptional elongation [100].

Several reports have described a physical and functional interaction between MALAT1 and the members of the PRC2 complex, EZH2, Suz12, and EED. Consistently, by facilitating binding of EZH2 to its target loci, MALAT1 drives H3K27 trimethylation and repression of tumor suppressor genes or microRNAs, leading to epithelial to mesenchymal transition, migration/invastion, cell proliferation, and escape from apoptosis in several tumor types [101, 102].

A further mechanism by which MALAT1 regulates transcription is provided by interactions with transcription factors. For instance, in MM mesenchymal stem cells, MALAT1 was found to activate the transcription of LTBP3 gene, located on the same MALAT1 locus on chromosome 11q13.1, by inducing the recruitment of Sp1 on LTBP3 promoter (Fig. 2c).

#### ceRNA function

The ceRNA function provides a further mechanism by which MALAT1 can post-transcriptionally control gene expression through inhibition of miRNAs [14]. By this mechanism, MALAT1 was proven to sequester miRNAs through miRNA responsive elements (MREs) located in its sequence, thus relieving the inhibitory effects of tumor suppressor miRNAs on oncogenic targets, and leading to phenotypic changes such as cell proliferation and invasion (Fig. 2d). Coherently with this function, MALAT1 promoted STAT3 expression by binding and sequestering its major negative regulator miR-125b in oral squamous cell carcinoma cells [103], and similarly triggered malignant melanoma growth and metastasis by sponging miR-22, and consequently upregulating the miR-22 targets MMP14 and Snail [104].

### Strategies for MALAT1 therapeutic targeting in cancer

A huge body of preclinical data indicates that MALAT1 is abundantly expressed in human neoplasias, and promotes proliferation and/or dissemination of tumor cells of various solid and hematological malignancies; consistently, meta-analyses have reported that elevated MALAT1 correlates with larger tumor size, advanced tumor stage and overall poor prognosis, and might therefore serve as biomarker to predict either clinicopathological features or prognostic outcome of cancer patients [105, 106].

On this basis, strategies for selective inhibition of MALAT1 have been developed to halt its oncogenic activity in a therapeutic perspective.

MALAT1 targeting in preclinical cancer models has been mainly achieved by the use of synthetic oligonucleotides. Although MALAT1 is located in the nucleus, and therefore is less accessible than mRNAs to siRNAs, several studies have reported its successful knockdown by RNA interference approaches [107]. Overall, inhibition of MALAT1 by siRNAs is achieved as the double-stranded RNA elicits a RISC-mediated degradation of the target lncRNA.

In addition, antisense oligonucleotides (ASOs) represent a valuable approach to antagonize MALAT1. ASOs are small oligonucleotides with RNA/DNA-based structures that selectively bind to RNA via Watson-Crick hybridization, capable to cross the cell membrane, and to bind the target RNA in the nucleus as well as in the cytoplasm [108].

Advantages of ASOs over siRNAs include their independence on the RISC machinery, higher specificity and fewer off-target effects. Most of the ASOs are double-stranded oligonucleotides using the RISC complex to mediate the degradation of RNA, or single-stranded ASOs that inhibit RNA function through different mechanisms, such as alteration of RNA splicing, activation of RNase H which degrades the target RNA, inhibition of 5′ cap formation and steric blockade of protein translation. Chemical modifications have been introduced to overcome some of the ASO constraints, like off-target/toxicity effects, high vulnerability to degradation by exo- and endonucleases, low affinity for the target, and poor delivery to the target tissues or low cellular uptake. These ASO modifications are defined as first generation and include a change in the phosphodiester with a phosphorothioate bond, the latter protecting the oligonucleotide from degradation and increasing the binding to receptor sites and plasma proteins [109]; conversely, second-generation modifications refer to changes in the sugar moiety of the nucleobase increasing binding affinity to the target [110]. The most relevant second-generation modification is the LNA (locked nucleic acid) [111]: this modification increases affinity, specificity and half-life determining effective delivery to target tissue with lower toxicity [108]. Finally, the third generation ASOs include peptide nucleic acid (PNA) and phosphorodiamidate morpholino oligomer (PMO) reporting modifications to the furanose ring of the nucleotide [110]. Depending on either the chemical modifications integrated into ASO or the type of inhibitory mechanism used, ASOs can be further divided in two main categories: mixmeRs and gapmeRs. In a mixmeR, LNA (locked nuclei acid) residues are dispersed throughout the ASO sequence, while gapmeRs contain a DNA or PS segment in the middle that promotes RNase H degradation of RNA target, flanked by LNAs [108]. Our group and others have demonstrated that LNA-inhibitors are efficient tools for targeting oncogenic microRNAs in preclinical models of human cancers [30, 112–122].

### MALAT1 role in cancer

MALAT1 expression and functional role in the onset and/or progression of different cancer types, along with the therapeutic potential of MALAT1 targeting strategies, will be below discussed. A summary of clinical features of tumors with elevated MALAT1 expression, along with MALAT1 molecular targets and phenotypic effects produced by its targeting in tumor preclinical models, is reported in Table 2.

**Table 2:** MALAT1-associated clinical and biological features of human cancer (↑ indicates increase; ↓ indicates decrease)

Tumor type	Associated clinical features of high MALAT1 tumors	MALAT1 molecular target(s)	Phenotypic effects induced by MALAT1 inhibition in preclinical models	Reference(s)
Non-small cell lung cancer	↓ survival of stage I NSCLC patients; ↓ overall survival; ↑ metastasis; ↑ in peripheral blood of NSCLC; ↑ in advanced tumor stages and in lymph-node metastasis	AIM1, LAYN, HMMR, SLC26A2, CCT4, ROD1, CTHRC1,FHL1, CXCL5	↓ migration and invasion in vitro; ↓ tumor growth in vivo; reduction or suppression of metastasis	[123–128]
Breast cancer	MALAT1-positive association with estrogen and progesterone receptors’ expression; ↓ recurrence-free survival in ER-negative patients	CD133, HuR; VEGF-A	↓ tumor growth and differentiation into cystic tumors; metastasis reduction; ↓ branching morphogenesis; ↑cell adhesion; ↓ migration	[129–132] [194]
Hepatocellular carcinoma	↑ risk of HCC recurrence after liver transplantation; ↑ in sera of arsenite-exposed people and of HCC patients; ↑ in III-IV TNM stages; ↓ overall survival of HCC patients	TRF2; p53; HIF2α	↓ in vitro and in vivo tumor growth	[78, 141–145]
Ovarian cancer	Correlation with FIGO stages; ↑ in peripheral blood correlating with distant metastasis	Matrix metalloproteinases; miR-506	↓cell proliferation, migration and invasion; G0/G1 cell cycle arrest; ↑ of apoptosis	[161–164] [195, 196]
Cervical cancer	↑ lymph-node metastasis; ↓ overall survival	Vimentin, β-catenin and E-cadherin	↓cell viability and proliferation in vitro and in vivo; ↓ migration and invasion	[135–139]
Esophageal cancer	↓ survival of patients undergoing radical resection of middle thoracic ESCC; positive correlation with pT stage and negative correlation with disease-free and overall survival; ↑ lymph nodes metastasis; ↓ disease-free survival	β-catenin; Lin28; EMT/stemness-related genes (OCT4, E-cadherin)	↓in vitro cell growth; ↓colony formation ability, migratory and invasive capabilities; G2/M phase cell cycle arrest and ↑ of apoptosis; ↓ tumor sphere formation; ↓ tumor formation in vivo	[147, 148]
Renal cell carcinoma	↓ overall survival	Twist, E-cadherin, EZH2, miR-200, ZEB2	↓cell proliferation and invasion	[169–172]
Prostate cancer	↑ of PSA-positive biopsies; ↑ in bone marrow of CRPC patients	Estrogen receptors (ERα/ERβ); EZH2	↓invasion and migration in vitro and in vivo	[158–160]
Osteoarcoma and Ewing sarcoma	↑ in serum correlating with worse overall survival; ↑ in tissues correlating with distant metastasis	E-cadherin, HMGB1; SYK-target genes	↓cell proliferation and migration, cell cycle arrest, ↑ apoptosis, both in vitro and in vivo	[79] [165–168]
Bladder cancer	↑ metastasis	EMT genes (E-cadherin, N-cadherin)	↓ cell migration and metastasis	[85] [177–179]
Brain cancer	Positive association with III-IV WHO grades and tumor size; ↑ MALAT1 in temozolomide-resistant patients; ↓ overall survival	miR-155; FBXW7; ERK/MAPK pathway; MMP2; thymidylate synthase	↓ tumor growth, migration and invasiveness in vitro; in vivo ↓ inhibition of tumor growth in vivo; restoration of temozolomide sensitivity; eradication of GBM stem cells	[180–186]
Endometroid endometrial carcinoma	Association with FIGO stages	PCDH10-Wnt/β-catenin; miR200c	↓ cell proliferation, migration and invasion	[76, 140]
Gastric cancer	↑ peritoneal metastasis; ↑ distant metastasis; ↓ overall survival of stage III and IV patients	miR-122/IGF-1R axis; PCDH10; miR-23b	↓ cell proliferation, cell cycle progression, migration and invasion; ↑ apoptosis; abrogation of chemoresistance	[149–153]
Colorectal cancer	↑ lymph-node metastases; ↓ overall survival	PRKA kinase anchor protein 9 (AKAP-9); CCL5; E-cadherin	Abrogation of EMT; reversion of oxaliplatin resistance	[173–176]
Pancreatic cancer	↑ overall survival; ↓ progression-free survival of patients undergoing gemcitabine-based chemotherapy as first-line treatment for locally advanced or advanced disease	Hippo-YAP1 pathway; P62, LAMP-2	↓ cell proliferation and migration, ↑ of apoptosis, in vitro and in xenograft murine models in vivo	[154–157]
Multiple myeloma	↑ MGUS, SMM, MM, and PCL; ↓ circulating MALAT1 in MM patients; ↑ MALAT1 in bone marrow mononuclear cells from MM patients	LTBP3; SP1; NRF1-NRF2/Keap1 pathway; proteasome-associated genes; miR-29b	↓ of in vitro and in vivo tumor growth and induction of apoptosis; ↓ clonogenicity; ↑ sensitivity to bortezomib	[27, 81, 101, 190]
Mantle cell lymphoma	↓ overall survival	p21 and p27 cell cycle inhibitors	↓ of cell proliferation, ↑ sensitivity to apoptosis; cell cycle arrest at G1/S transition	[192]
T cell lymphoma	↓ overall survival	PRC2 components	–	[193]

### Solid tumors

#### Lung cancer

MALAT1 is an adverse prognostic marker both in stage I lung adenocarcinoma and squamous cell carcinoma patients, and its expression also associated with metastasis [65, 123]. Tano et al. established a link between MALAT1 and transcriptional and/or post-transcriptional modulation of genes regulating cell motility, including AIM1, LAYN, HMMR, SLC26A2, CCT4, ROD1, CTHRC1, and FHL1. Schmidt then demonstrated that siRNA-mediated knockdown of MALAT1 significantly impaired migration and invasion in vitro [124], as well as in vivo tumor growth in mice. The key role of MALAT1 in metastasis was indeed disclosed by Gutschner et al., who showed that MALAT1 knock-out clones achieved by zinc finger nuclease-mediated site-specific integration of RNA destabilizing elements into the genome of lung cancer cells, failed to induce distant metastasis [125]. Mechanistically, it was demonstrated that MALAT1 modulated migration and invasion of non-small cell lung cancer (NSCLC) cells by targeting the epithelial-derived neutrophil-activating peptide belonging to the CXC chemokine family (CXCL5). The same authors showed that MALAT1 is regulated by epigenetic mechanisms, since methylation of CpG islands at MALAT1 promoter decreased in cancer as compared to normal tissues [126]. Noteworthy, Jen et al. provided evidence of transcriptional control of MALAT1 expression in lung cancer by the stemness transcription factor OCT4, which bound to MALAT1 enhancer regions and triggered its expression, thus enhancing proliferation, migration and invasion of tumor cells in vitro. Of potential clinical significance, high levels of OCT4/MALAT1 correlated with poor outcome of patients [127].

Experimental strategies targeting MALAT1 with second generation of naked ASOs demonstrated drastic reduction of lung cancer metastasis in a pulmonary metastatic model in vivo, making MALAT1 a suitable target for anti-metastatic therapy [125].

Recently, it has been also shown that MALAT1 is highly expressed in exosomes released by NSCLC cells and the levels of exosomal MALAT1 positively associated with tumor stage and lymphatic metastasis of NSCLC patients [128].

#### Breast cancer

MALAT1 overexpression in breast cancer (BC) has been reported by different research groups [129, 130]. In triple-negative breast cancer (TNBC) cells, the lysine-specific demethylase 5B (KDM5B) promoted MALAT1 expression, which in turn enhanced the invasion and clonogenic potential in in vitro and in vivo models [92]. Tumor epithelial cells overexpressing MALAT1 showed marked diffuse nuclear signals with numerous nuclear speckles [129]. Genetic loss or systemic delivery of ASOs targeting MALAT1 in the mouse mammary tumor virus-PyMT (MMTV) carcinoma model resulted in slower tumor growth, significant differentiation into cystic tumors and metastasis reduction; furthermore, MALAT1 loss decreased branching morphogenesis in MMTV-PyMT and Her2/neu-amplified tumor organoids, increased cell adhesion, and reduced migration. At the molecular level, MALAT1 knock-down halted the expression of splicing genes involved in differentiation and pro-tumorigenic pathways [131].

A potential role of MALAT1 in the breast stem cell compartment was proposed by Latorre et al., who demonstrated that MALAT1 regulated the stem cell marker CD133 via the interaction with the RNA-binding protein HuR (ELAVL1); HuR silencing in luminal non-metastatic breast cancer cells was sufficient to upregulate N-cadherin (CDH2) and CD133, thus leading to a mesenchymal-like and migratory phenotype. In the basal-like metastatic cell line MDA-MB231 and in primary TNBC cells, MALAT1-HuR repressor complex was absent from the CD133-regulatory region, and this was attributed to diminished expression of MALAT1, which, when overexpressed, decreased CD133 levels [132]. In BC cells, oncogenic splicing factor SRSF1 bridges MALAT1 to mutant p53 and ID4 proteins, favoring its chromatin association and thus inducing the expression of various VEGF-A isoforms, indicating a role of MALAT1 in the promotion of angiogenesis [133].

Interestingly, interrogation of dbEST database allowed the identification of an alternatively spliced MALAT1 transcript (Dsv-MALAT1) that was mainly underexpressed in breast tumor samples as compared to full length MALAT1, and which acted as an independent prognostic factor. Noteworthy, Dsv-MALAT1 expression associated with alterations of pre-mRNA alternative splicing machinery, and with the expression of the Drosha-DGCR8 complex required for miRNA biogenesis [129].

Surprisingly, recent work by Kwok et al. unveiled a tumor suppressive role of MALAT1 in BC and colorect cancer, where MALAT1 was induced by the tumor suppressor PTEN and negatively affected the expression of genes implicated in migration and invasion, such as EpCAM and ITGB4 [134].

#### Cervical cancer

MALAT1 transcript was upregulated in cervical cancer (CC) as compared to normal cervix, and its expression correlated with worse overall survival in CC patients and associated with lymph-node metastasis [135]. Jiang and colleagues disclosed a direct correlation between MALAT1 and HPV in CC cell lines, suggesting that MALAT1 could be a potential biomarker for screening [136]. Loss of function studies by siRNAs demonstrated the tumor promoting role of MALAT1 in CC cell viability, proliferation, migration, and invasion both in vitro and in vivo [135, 137]. MALAT1 also regulated EMT in CC, as demonstrated by the decrease in EMT-related genes Vimentin and β-catenin, and the upregulation of E-cadherin gene upon MALAT1 silencing [138]. Furthermore, MALAT1 was linked with miR-375 in a feedback loop ultimately triggering EMT in CC cells. Indeed, the authors demonstrated that miR-375 targeted MALAT1, partly restored E-cadherin levels, and significantly reduced N-cadherin and the invasive capability of CC cells [139].

#### Endometroid endometrial carcinoma

Zhao et al. investigated the molecular mechanisms implicating MALAT1 in endometrial tumorigenesis and identified MALAT1 as a downstream target of the protocadherin10 (PCDH10) gene, a Wnt pathway negative regulatory element, shedding light on a novel targetable PCDH10-Wnt/β-catenin-MALAT1 axis [76]. In a recent study, MALAT1 was found targeted by miR-200c, which plays a negative role in tumor growth and EMT; accordingly, an inverse correlation between MALAT1 and miR-200c levels emerged in EEC tissues. Interestingly, authors found that strategies targeting the miR-200c/MALAT1 axis, mainly by miR-200 mimics or MALAT1 shRNAs, significantly impaired cell migration, invasion, and in vitro and in vivo EEC growth [140].

#### Hepatocellular carcinoma

MALAT1 is overexpressed in hepatocellular carcinoma (HCC) primary samples and cell lines [141], and its expression correlated with advanced tumor stages and reduced overall survival of HCC patients [78]; moreover, high MALAT1 levels correlated with major risk of HCC recurrence after liver transplantation [142]. Mutational signatures related to liver carcinogenesis, including MALAT1 and NEAT1 lncRNAs, were found in the japanese population [143], although their clinical significance remains unexplored.

Interestingly, the carcinogen arsenite could transcriptionally induce MALAT1 via HIF2α, and MALAT1 was found upregulated in the sera of people exposed to arsenite as well as in HCC patients; in turn, MALAT1 was able to promote the disassociation of the von Hippel-Lindau (VHL) protein from HIF-2α, therefore alleviating VHL-mediated HIF-2α ubiquitination and degradation. Importantly, lentiviral shRNA targeting of MALAT1 reduced tumor growth in vivo in xenograft models of arsenite-transformed HCC cells, strengthening the role of MALAT1 in carcinogen-induced tumorigenesis [78].

Recently, Wang and colleagues [144] highlighted a novel mechanism by which MALAT1 is upregulated in HCC: indeed, they showed that the Yes-associated oncoprotein YAP1 increased MALAT1 at both transcriptional and post-transcriptional level, thus accelerating HCC proliferation. Moreover, Wu et al. highlighted a functional interaction between MALAT1 and the lncRNA HULC-1, both overexpressed in HCC; such interaction was instrumental in promoting growth of liver cancer stem cells through induction of telomere repeat-binding factor 2 (TRF2) telomerase activity [145]. By elegant mechanistic studies performed in HepG2 cells, it was further shown that MALAT1 competed with Sirt1 deacetylase for the binding with DBC1 (depleted in breast cancer 1), thus releasing Sirt1 and enhancing its deacetylase activity. As a consequence, p53 was deacetylated and did not transactivate its pro-apoptotic target genes, leading to uncontrolled cell growth [146].

#### Esophageal squamous cell carcinoma

Different studies reported that MALAT1 is overexpressed in Esophageal squamous cell carcinoma (ESCC). Yao et al. first demonstrated that MALAT1 was highly increased in tumor lesions as compared to non-cancerous tissues, and correlated with shorter overall survival rate of ESCC patients. In vitro studies showed that siRNA-mediated depletion of MALAT1 decreased cell growth, the colony formation ability, as well as the migratory and invasive capabilities of ESCC cells, along with G2/M cell cycle arrest and apoptosis induction [147]. Additionally, MALAT1 could be considered a predictive marker of poor prognosis in patients undergoing radical resection of middle thoracic ESCC, since its expression positively correlated with pT stage, and patients with high MALAT1 levels had a higher T stage and a shorter disease-free and overall survivals [148].

Recently, Wang et al. confirmed MALAT1 overexpression in human ESCC as compared to matched adjacent non-cancerous tissues, with the highest expression in stages III-IV patients, as well as in lymph node metastasis.

In vitro studies revealed that knock-down of MALAT1 reduced cell proliferation, migration and number of tumor spheres representing putative stem cell-like cells, along with increasing cell apoptosis. Moreover, MALAT1 downregulation induced a decrease of β-catenin, Lin28, EZH2 and EMT stem genes as OCT4, while promoted E-cadherin expression. Of note, EZH2 overexpression completely reverted repression of β-catenin and Lin28 induced by MALAT1-targeting siRNAs, thus supporting EZH2-dependent activity of MALAT1 in ESCC.

In vivo studies showed that siRNA-mediated depletion of MALAT1 reduced tumor formation in mice and enhanced animal survival, supporting is potential as therapeutic target [102].

Interestingly, tumor suppressor miR-101 and miR-217 acted as post-transcriptional regulators of MALAT1, whose silencing induced cell cycle arrest by p21 and p27 upregulation, and b-MYB inhibition. miR-101 and miR-217 overexpression or MALAT1 knockdown via miRNA mimics and siRNAs respectively, inhibited migration and invasion capabilities of ESCC cells, and were accompanied by deregulation of MALAT1 downstream metastasis-associated genes MIA2, HNF4G, ROBO1, CCT4, and CTHRC1 [88].

#### Gastric cancer

There is a wealth of evidences about MALAT1 overexpression in gastric cancer (GC). Okugawa et al. reported that MALAT1 was significantly higher in GC tissues and cell lines respect to normal mucosa, and it significantly correlated with peritoneal metastasis in GC patients [149]. In addition, since tissue and plasma MALAT1 levels scored higher in GC patients with distant metastasis, MALAT1 could likely serve as a potential biomarker for GC metastases. Importantly, high plasma levels of MALAT1 independently correlated with poor prognosis of GC patients. In GC clinical specimens, MALAT1 expression was tightly associated with densities of endothelial vessels, and with vasculogenic mimicry [150]. Moreover, knock-down of MALAT1 in GC cells inhibited cell proliferation, cell cycle progression, migration and invasion, and promoted apoptosis. Mechanistically, miR-122-IGF-1R signaling was found involved in dysregulated MALAT1 expression. Indeed, enforced miR-122 expression inhibited, whereas miR-122 inhibitor increased MALAT1 in GC cell lines. miR-122-mediated regulation of MALAT1 involved IGF-1R, a target of miR-122, whose expression positively correlated with MALAT1 in GC cell lines [151]. Furthermore, Qi et al. confirmed MALAT1 overxpression in GC cell lines, where it promoted cellular migration and invasion by epigenetic effects involving EZH2 and suppression of the tumor suppressor protocadherin 10 (PCDH10). High expression of MALAT1 was associated with poorer overall survival of stage III and IV GC patients [152], thus underlining the prognostic power of MALAT1 in GC.

MALAT1 involvement in GC chemo-resistance was demonstrated by YiRen et al., who found high levels of MALAT1 in vincristine-resistant cells, where MALAT1 mechanistically promoted pro-survival autophagy. MALAT1 silencing by shRNAs sensitized GC cells to chemotherapeutics by upregulating miR-23b, a miRNA sponged by MALAT1, which suppressed chemo-induced autophagy and related chemoresistance [153].

#### Pancreatic cancer

By interrogating Gene Expression Omnibus, Oncomine, and The Cancer Genome Atlas databases, authors found MALAT1 significantly elevated in patients with pancreatic cancer, and ROC curves showed a moderate diagnostic power of MALAT1 expression. Interestingly, serum levels of MALAT1 and other two lncRNAs, PVT1, and HOTTIP, acted as biomarkers predicting efficacy of gemcitabine-based chemotherapy as first-line treatment for locally advanced or advanced pancreatic cancer, with progression-free survival of patients with high and low MALAT1 expression levels being 3.0 and 3.7 months, respectively [154].

Several pathways, including mTOR and MAPK, were found to mediate MALAT1 oncogenic activity in PC, as predicted by in silico analyses [128, 155]. MALAT1 silencing by siRNAs antagonized tumor cell proliferation and migration, and triggered apoptosis, both in vitro and in xenograft murine models in vivo. At the molecular level, inhibitory effects on cell proliferation were also linked either to the blockade of the Hippo-YAP1 pathway, which is hyperactivated in multiple types of cancer [156], or to the suppression of cytoprotective autophagy [157].

#### Prostate cancer

MALAT1 was found overexpressed in prostate cancer (PCa), where it served as diagnostic urinary biomarker for predicting risk of PCa. MALAT1 score, defined as the ratio between MALAT1 and PSA mRNAs, was significantly higher in men with positive biopsy, and allowed to define a MALAT1-based model likely preventing 30.2–46.5% of unnecessary biopsies in PSA 4–10 ng/ml cohorts with a probability threshold of 25% [158]. In PCa cells and organotypic slice cultures of organ-confined prostate tumors, MALAT1 and the lncRNA HOTAIR were implicated in estrogen-mediated transcriptional regulation. Specifically, both MALAT1 and HOTAIR were regulated by estrogens, and controlled estrogen receptors’ function by interacting with ERα/ERβ; such interaction appeared necessary for a complete estrogen signaling both in vitro and in vivo. Upon treatment with 17β-estradiol, chromatin recruitment of HOTAIR increased, while that of MALAT1 decreased, indicating an opposite regulation and function for these two lncRNAs [159]. In addition, MALAT1 was highly expressed in bone marrow biopsy specimens of castration-resistant prostate cancer (CRPC) [160]. In line with an epigenetic role, Wang and colleagues identified MALAT1 as a regulator of EZH2 in CRPC cells, as MALAT1 interacted with and facilitated promoter occupancy and H3K27me3 activity of EZH2; furthermore, MALAT1 facilitated EZH2-mediated PCa cell invasion and migration and enhanced expression of EZH2 target genes [158].

#### Ovarian cancer

As shown by Zhou et al., MALAT1 is upregulated in ovarian cancer (OC) tissues, and its expression correlated with International Federation of Gynecology and Obstetrics (FIGO) stages. In vitro, MALAT1 overexpression prompted OC proliferation, migration and invasion, while its depletion led to the opposite effects, and also increased CDDP-sensitivity [161–163]. Interestingly, MALAT1 affected the expression of genes of the matrix metalloproteinase (MMP) family involved in extracellular matrix metabolism: specifically, MALAT1 knock-down caused upregulation of MMP13, and downregulation of MMP19 and metallopeptidase with thrombospondin type-1 motif (ADAMTS1) [161]. Furthermore, Lei et al. observed MALAT1 upregulation in OC cell lines and specimens, and identified a novel mechanism by which MALAT1 enhanced tumor cell growth by targeting miR-506 in the context of a negative feed-back loop.

Authors also disclosed a positive correlation between the expression of MALAT1 and iASPP, a member of the apoptosis-stimulating proteins of p53 (ASPP) family [164].

#### Sarcoma

MALAT1 is frequently upregulated in osteosarcoma (OS) primary tissues and cell lines, and the analysis of clinical samples demonstrated correlation of high serum levels of MALAT1 with reduced survival rate in OS patients [165]. MALAT1 was shown to promote OS cell growth and metastasis by different mechanisms. Huo et al. showed that MALAT1 transcription was activated by TGF-β; additionally, MALAT1 interaction with the PRC2 member EZH2 was capable to suppress E-cadherin expression, thus promoting OS metastasis [166]. Additionally, Liu et al. demonstrated that MALAT1 promoted OS cell growth by inducing HMGB1 activity through inhibition of its negative regulators miR-142-3p and miR-129-5p. Consistent with all these findings, siRNA-mediated knockdown of MALAT1 inhibited cell proliferation and migration, and induced cell cycle arrest and apoptosis in OS cells, both in vitro [167] and in vivo [168].

In Ewing sarcoma (EWS), a devastating soft tissue sarcoma affecting predominantly young individuals, MALAT1 was identified to be transcriptionally upregulated through a SYK/c-MYC axis, and to positively mediate SYK tyrosine kinase oncogenic activity [79].

#### Renal cell carcinoma

MALAT1 is frequently overexpressed in renal cell carcinoma (RCC) cell lines and primary tumor samples as compared to normal tissues; moreover, high MALAT1 expression associated with worse overall survival in RCC patients [169]. Intriguingly, fusion of the TFEB gene on chromosome 6p21.2 and MALAT1 gene on chromosome 11q13 was observed in RCC, and likely associated to favorable clinical prognosis [170].

In RCC cells, MALAT1 was positively regulated by VHL pathway through c-FOS transcription factor and promoted EMT features of tumor cells in a PRC2-dependent manner, since EZH2 depletion inhibited MALAT1-dependent tumor-promoting activity [171]. Interestingly, MALAT1 positive effects on EMT were further ascribed to a ceRNA mechanism: through miR-200 sponging, MALAT1 upregulated zing finger E-box-binding homeobox 2 (ZEB2), which in turn promoted proliferation and dissemination of RCC cells [172].

#### Colorectal cancer

MALAT1 was found upregulated in human primary colorectal cancer (CRC) tissues with lymph node metastasis. Clinically, high levels of MALAT1 and other lncRNAs, including AFAP1-AS1, BCAR4, H19, HOXA-AS2, and PVT1, were predictive of poor prognosis of CRC patients [173]. MALAT1 overexpression promoted CRC cell proliferation, invasion and migration in vitro, and stimulated tumor growth and metastasis in mice [174]. By genome-wide profiling, Yang et al. showed that among 243 genes regulated by MALAT1 in CRC cells, PRKA kinase anchor protein 9 (AKAP-9) was significantly upregulated at both mRNA and protein level. Of note, knockdown of AKAP-9 rescued MALAT1-induced CRC cell proliferation, migration and invasion. MALAT1 was also associated with colon cancer progression by stimulating tumor associated dendritic cells (TADC)-derived production of CCL5; consistently, MALAT1 blockade by siRNAs significantly dampened CCL5-induced migration and invasion of CRC cells [175].

Importantly, MALAT1 was shown to induce oxaliplatin-resistance partly through suppression of E-cadherin signaling and induction of EMT, acting in an EZH2-dependent manner; consistently, targeted inhibition of both MALAT1 and EZH2 via siRNAs reversed EMT features and restored oxaliplatin sensitivity [176].

#### Bladder cancer

Li et al. showed that high MALAT1 expression could serve as an independent prognostic factor for overall survival of patients with bladder cancer, and could be considered a potential therapeutic target [177]. Moreover, Ying and colleagues showed that MALAT1 promoted bladder cancer cell migration and metastasis by inducing EMT [178]. Mechanistically, MALAT1 association with PRC2-component Suz12 led to reduction of E-cadherin and induction of N-cadherin, triggering EMT in vitro and tumor growth and dissemination in vivo [85].

Serum MALAT1 was also found upregulated in bladder cancer patients compared to healthy individuals and could likely a represent a novel independent biomarker for the diagnosis or recurrence of this cancer [179].

#### Brain cancer

Ma et al. showed that MALAT1 is downregulated in glioma, where high expression correlated with improved survival in patients [180]. Interestingly, preliminary reports showed that MALAT1 acted as tumor-suppressor gene, thus suggesting that its restoration might be a novel therapeutic approach against glioma. In this light, Cao and colleagues demonstrated that MALAT1 inhibited cell viability by down-regulating miR-155 and promoting FBXW7 expression, a tumor suppressor promoting the degradation of substrates with oncogenic activity such as Cyclin E, c-Myc and AURKA [181]. Moreover, Han et al. revealed that upregulation of MALAT1 significantly reduced cell growth by inhibiting ERK/MAPK pathway and MMP2-mediated invasiveness both in vitro and in vivo [182]. Conversely, Vassallo et al. revealed that WNT inhibitory factor 1 WIF1 down-modulated MALAT1, whose expression in turn enhanced migration of tumor cells both in vitro and in vivo [183].

By deep sequencing analysis of temozolomide-sensitive and resistant patient glioma cells, MALAT1 was shown to discriminate responding from non-responding patients; moreover, MALAT1 was found to promote in vitro chemoresistance through miR-203 suppression, which is involved in cell proliferation pathway through thymidylate synthase targeting [184]. Additionally, by sponging miR-101, MALAT1 abolished miR-101-dependent negative regulation of the autophagic program in glioma cells, thus prompting cell proliferation [185].

Importantly, targeted nanocomplexes carrying MALAT1-targeting siRNAs were able to eradicate glioblastoma stem cells, leading to improved sensitivity of tumor cells to temozolomide in animal models of glioblastoma [186].

### Hematological malignancies

#### Multiple myeloma

Cho et al. found MALAT1 overexpressed in bone marrow mononuclear cells from newly-diagnosed multiple myeloma (MM) patients as compared to treated patients or healthy individuals. Moreover, patients who experienced disease progression or relapse showed an increased expression of MALAT1; of note, MALAT1 expression in newly diagnosed patients did not correlate with the percentage of plasma cells in the bone marrow [187]. Our group and others recently confirmed progressive increase of MALAT1 levels from normal plasma cells to overt MM, and further increase in the extramedullary phases, in a large number of clinically-annotated patients [27] [188]. On the other hand, expression of circulating MALAT1 was significantly lower in MM patients [189].

Regarding MALAT1 mechanism of action, Ronchetti et al. showed that upregulation of MALAT1 in MM associated with molecular pathways regulating cell cycle, p53-mediated DNA damage response, and mRNA maturation processes. Furthermore, Li et al. demonstrated that MALAT1 regulates the transcription of the neighboring antisense protein-coding gene LTBP3, a crucial regulator of bone formation, in mesenchymal stem cells from MM patients, with potential implications for MM-related bone disease. At the molecular level, investigators reported that the transcription factor SP1 is recruited by MALAT1 on the LTBP3 promoter, promoting an increase of LTBP3 expression [190]. Importantly, selective targeting of MALAT1 by LNA gapmeR ASOs triggered apoptosis in vitro and in vivo in a murine model of human MM and also overcame the protective bone marrow microenvironment, which is known to promote survival and drug resistance of MM cells. Intriguingly, MALAT1 ASO-knock-down inhibited all proteasome activities (trypsin-like, chymotrypsin-like, and caspase-like) by targeting the well-established positive regulators of proteasome gene expression NRF1 and NRF2, through EZH2-mediated regulation of the NRF1/2 negative regulator KEAP1 [81].

Interaction between EZH2 and MALAT1 also impacted the miRNome, since inhibition of either MALAT1 or EZH2 via LNA gapmeR ASOs and small molecule EZH2 inhibitors respectively, reduced H3K27me3 repressive marks at miR-29a/b-1 promoter [101], with consequent upregulation of miR-29b, a relevant tumor suppressive miRNA in MM [30, 191].

#### Lymphomas

MALAT1 is abundantly expressed and associated with reduced overall survival of mantle cell lymphoma (MCL) patients. Of note, MALAT1 targeting by siRNAs blocked cell proliferation and increased apoptotic cell death. As seen in other neoplasias, Wang and colleagues also underscored an EZH2-mediated epigenetic activity of this lncRNA in MCL, since MALAT1 knock-down reduced EZH2 levels and decreased H3K27me3 at the promoter of the target genes p21^WAF1^ and p27^KIP1^, leading to cell cycle arrest at G1/S phase [192]. MALAT1 was also found to interact with, and to positively correlate with the expression of PRC2-components EZH2 and SUZ12, in NK and T cell lymphomas [193].

## Conclusions

Multiple lines of evidence have reported aberrant expression and prognostic usefulness of tissue MALAT1 across several tumor types, along with diverse and context-dependent molecular mechanisms leading to the acquisition of the malignant phenotypes.

To date, the complex tridimensional structure of lncRNAs allowing interaction with many RNA and/or protein partners, along with the resultant pleiotropic mode of action, have slowed down acquisitions on lncRNA functions in normal and pathological contexts. In this regard, novel biochemical approaches to study lncRNAs are progressively emerging, and will likely disclose unknown functions of lncRNAs, including MALAT1, hopefully widening the spectrum of biological activities and the precise role in disease pathobiology.

Significant advances have been also achieved in developing therapeutic reagents for drugging oncogenic lncRNAs in tumor cells, and novel approaches are being addressed to design and develop small molecules targeting lncRNAs within their tridimensional conformations.

Overall, targeted genetic deletion of MALAT1 by zinc finger nucleases, as well MALAT1 therapeutic targeting by synthetic oligonucleotides, including siRNAs and the newly developed LNA gapmeR ASOs, have established the oncogenic role of this lncRNA and its druggability for therapeutic purposes.

Although additional studies with more sophisticated and physiologically relevant in vivo models recapitulating certain types of cancers are undoubtedly required, available findings indeed point to MALAT1 as lead candidate for novel clinically translatable lncRNA-based therapeutic strategies against cancer.

## Abbreviations

AFAP1-AS1: AFAP1-antisense RNA
AIM1: Absent in melanoma 1
AKAP-9: A-Kinase anchoring protein 9
AMACAR: Alpha-methylacyl-CoA racemase
BCAR4: Breast cancer anti-estrogen resistance 4
BCL2: B cell lymphoma 2
BDNF-AS1: Brain-derived neurotrophic factor antisense 1
CC: Cervical cancer
CCAT1: Colon cancer-associated transcript 1
CCL5: Chemokine C-C motif ligand 5
CCT4: Chaperonin Containing TCP1 subunit 4
CD133: Cluster of differentiation 133
CDKN1A: Cyclin-dependent kinase inhibitor 1A
CDKN2B: Cyclin-dependent kinase inhibitor 2B
ceRNA: Competing endogenous RNA
c-FOS: FBJ osteosarcoma oncogene
CRC: Colorectal cancer
CRPC: Castration-resistant prostate cancer
CTHRC1: Collagen triple helix repeat containing 1
CXCL5: C-X-C motif chemokine ligand 5
DLEU: Deleted in leukemia
DSCAM-AS1: Down syndrome cell adhesion molecule antisense RNA 1
EEC: Endometroid endometrial carcinoma
EMT: Epithelial-to-mesenchymal transition
ER: Estrogen receptor
ERK/MAPK: Extracellular signal-regulated kinase/mitogen-activated protein kinase
ESCC: Esophageal squamous cell carcinoma
EZH2: Enhancer of zeste homolog 2
FBXW7: F-Box and WD repeat domain containing 7
FHL1: Four and a half LIM domains 1
GC: Gastric Cancer
H3K27me3: Histone H3 lysine 27 trimethylated
H3K36me2: Histone H3 lysine 36 dimethylated
HCC: Hepatocellular carcinoma
HMMR: Hyaluronan-mediated motility receptor
hnRNP-k: Heterogeneous nuclear ribonucleoprotein k
HOTAIR: HOX transcript antisense RNA
HOXA-AS2: HOXA cluster antisense RNA 2
HOXB13: Homeobox B-13
HULC-1: Hepatocellular carcinoma upregulated long non-coding RNA
HuR: Hu antigen R
iASPP: Inhibitor of apoptosis-stimulating protein of p53
IGF-1R: Type I insulin-like growth factor receptor
JMJD1A: Jumonji C-domain-containing protein 1A
LAYN: Layilin
lincRNA: Long intergenic non-coding RNA
LNA ASO: Locked nucleic acid antisense oligonucleotide
lncRNA: Long non-coding RNA
LTBP3: Latent transforming growth factor beta-binding protein 3
MALAT1: Metastasis associated lung adenocarcinoma transcript 1
miRNA: MicroRNA
MMP: Matrix metalloproteinase
MMTV: Mouse mammary tumor virus-PyMT
MREs: miRNA response elements
NALT: Notch1 associated lncRNA in T-ALL
ncRNA: Non coding RNA
NEAT1: Nuclear-enriched abundant transcript 1
NF-YA: Nuclear transcription factor Y subunit alpha
NK: Natural killer
NSCLC: Non-small cell lung cancer
OCT4: Octamer-binding transcription factor 4
OS: Osteosarcoma
PCa: Prostate cancer
PCAT-1: Prostate cancer-associated transcript 1
PCDH10: Protocadherin10 gene
piRNA: Piwi-interacting RNA
PRC2: Polycomb repressive complex 2
PSA: Prostate specific antigen
PSMA: Prostate-specific membrane antigen
PVT1: Plasmacytoma variant translocation gene 1
RCC: Renal cell carcinoma
ROD1: Regulator of differentiation 1
SET: SET nuclear proto-oncogene
siRNA: Small interfering RNA or short interfering RNA or silencing RNA
SIRT1: Sirtuin-1
SLC26A2: Solute carrier family 26 member a2
snoRNA: Small nucleolar RNA
SPRY4: Sprouty RTK signaling antagonist 4
SR: Serine- and arginine-rich
Suz12: Suppressor of zeste 12 protein homolog
TADC: Tumor-associated dendritic cells
T-ALL: T-acute lymphoblastic leukemias
TDP43: TAR DNA-binding protein 43
TNBC: Triple-negative breast cancer
TRF2: Telomere repeat-binding factor 2
VHL: Von Hippel-Lindau
WHO: World Health Organization
WIF1: WNT inhibitory factor 1
YAP: Yes-associated protein
ZEB2: Zing finger E-box-binding homeobox 2
